# *Berberis* Fruit Extract and Biochemical Parameters in Patients With Type II Diabetes

**DOI:** 10.17795/jjnpp-13490

**Published:** 2014-04-07

**Authors:** Zolikha Moazezi, Durdi Qujeq

**Affiliations:** 1 Department of Internal Medicine, Babol University of Medical Sciences, Babol, IR Iran; 2 Cellular and Molecular Biology Research Center, Babol University of Medical Sciences, Babol, IR Iran; 3 Department of Biochemistry and Biophysics, Babol University of Medical Sciences, Babol, IR Iran

**Keywords:** Berberis, Diabetes Mellitus, Hemoglobin A, Glycosylated

## Abstract

**Background:**

Diabetes mellitus is a common medical problem. There is in fact a growing body of literature on plants used for the treatment of diabetes. Plant materials attracted considerable interest of scientists. In this respect, in the past few years, attempts were made to use natural plant products for the treatment of patients with diabetes.

**Objectives:**

The aim of this study was to investigate the effect of *Berberis* fruit extract to achieve glycemic control in patients with Type II diabetes.

**Materials and Methods:**

This study was performed between July 2010 and April 2013. Thirty patients of type II diabetes admitted to Ayatollah Rohhani hospital were recruited. Patients’ sera were collected for the assessment of glucose and HbA1c values. Biochemical analyses were performed before and after treatment by *Berberis* fruit extract. Biochemical parameters were measured by spectrophotometric method (Jenway uv/vis, 6505 model, Dunmow, UK). Glucose level was measured by glucose oxidase method kit (Pars Azmoon, Tehran, IR Iran). Serum total cholesterol and triglycerides were measured using standard biochemical kits (Pars Azmoon, Tehran, Iran). Blood glycated hemoglobin level was measured by using Elisa kit (Bioassay technology laboratory, Elisa kit). Experiments were performed in triplicate in at least three separate experiments.

**Results:**

Our findings demonstrated that patients with type 2 diabetes who received barberry fruit had significant reduction in serum glucose to 136.15 ± 32.8 mg/dL and decreased HbA1c levels to 7.07 ± 1.21 mg/dL, during the 8 weeks of study.

**Conclusions:**

This investigation revealed that *Berberis* fruit extract has beneficial metabolic effects in patients with type II diabetes. Barberry may improve glucose catabolism via glycolysis pathway, stimulate insulin secretion or improve insulin function and finally decrease glucose uptake. Our results indicated that *Berberis* fruit regulates glucose metabolism in patients with type 2 diabetes.

## 1. Background

 Major types of diabetes mellitus include type -I and type-II diabetes. Type-II insulin-resistant diabetes mellitus accounts for 90-95% of all diabetes. Previous studies demonstrated that hyperglycemia is the major component of metabolic dysfunction in type 2 diabetes (1, 2). In this respect, a number of recent studies have suggested that Berberine can reduce body weight and improve glucose tolerance in mice and rat models (3). There are some reports in the literature showing that Berberine may increase glucose-stimulated insulin secretion (4). Furthermore, preliminary studies revealed that *Berberis aristata* was found to lower blood glucose in alloxan induced diabetic rats, reduce oxidative stress and modulate enzymes responsible for glucose metabolism (5). As reported by investigators, the plant fruits have been also used as food additive (6). As previously demonstrated, Berberine is a plant alkaloid used in traditional Chinese medicine and has been reported to have anti-hyperglycemic property in patients with type II diabetes (7). It was shown that Berberine, an alkaloid isolated from bark and root of *Berberis vulgaris*, exerted an anxiolytic effect in mice (8). Several experimental studies have reported that *Berberis vulgaris* fruit (barberry) is known for its anti-arrhythmic and sedative effects (9). Recent studies demonstrated that Berberine stimulates glucose metabolism via stimulation of glycolysis, which is related to inhibition of glucose oxidation in mitochondria (10). On the other hand, some recent evidences indicated that Berberine inhibits citric acid cycle pathways (11-13). Preliminary studies conducted by many investigators revealed that Berberine improves insulin function (14). Several experimental studies indicated that Berberine reduces insulin resistance (15). In the recent years, laboratory studies suggested that Berberine may have at least two functions regarding lowering the blood sugar, inhibiting absorption of sugars from the intestine and enhancing production of insulin (16). Recent studies have suggested that Berberine regulates glucose metabolism. The hypoglycemic effect of Berberine was similar to that of lowering glucose medications. Significant decreases in hemoglobin A1c were observed (17). Besides, Hb-Alc level was increased in patients with diabetes mellitus as a consequence of increased blood glucose contents (18). Given the above findings the recent clinical interest in to study the possible change level of HbA1c in patients with diabetes mellitus. On the other hand, it is interesting that glycosylated hemoglobin concentration has been suggested as an index of control in the management of diabetes (19-22).

## 2. Objectives

The goal of this study was to investigate the effect of *Berberis* fruit on blood glucose level in patients with type II diabetes. 

## 3. Materials and Methods

### 3.1. Preparation of Extracts

*Berberis* fruit (barberry) was collected from Larijan, located near Amol, Iran in 2010. *Berberis* fruits extract was subjected to optical spectra. Optical spectra of barberry fruit was recorded by using a spectrophotometer (Jenway uv/vis, 6505 model, Dunmow, UK) with one milliliter cuvettes. *Berberis* fruit extraction was performed based on a previously described method (5, 6, 8). The extracts were filtered and the dry matter was maintained desiccated at 5˚C until use, as described before (16, 17). The same batch of extract was used in all the experiments. *Berberis* fruit was dried at room temperature and then powdered. To prepare powder concentrate, 1mg of dry *Berberis* fruit was put in capsule; totally, 2000 capsules were prepared.

### 3.2. Patients

The subjects of this study were patients admitted to medical center of Ayatollah Rohhani Hospital of Babol University of Medical Sciences from July 2010 to April 2013. This was a 2-month randomized, double-blind, and placebo-controlled study. Placebo was standard capsule without *Berberis* fruit. Written informed consent was obtained from all participants. No blood samples were drawn for the study unless an informed consent form was signed. The registration number was IRCT 3808, April 25, 2010 (clinical trials). Initial screening included a medical history, physical examination, serum glucose and lipid concentrations. During the 8-week run-in period, 30 patients with diagnosed type 2 diabetes were recruited. Seventeen patients were excluded due to not completing their follow up visits. The remaining 13 patients were randomly assigned to receive double-blind *Berberis* fruit or placebo.

### 3.3. Inclusion and Exclusion Criteria

Inclusion criteria were as follows:

1. Patients aged 30 to 65 years;
2. diagnosed type 2 diabetes, plasma glucose greater than 7 mmol/L;
3. dyslipidemia with triglycerides greater than 170 mg/dL, and total cholesterol greater than 220 mg/dL.

A standardized interview was conducted by trained personal regarding lifestyle habits, including smoking, physical activity and diet. The exclusion criteria were as follows:

1. a history of liver dysfunction, including serum alanine aminotransferase greater than 120 IU/L, aspartate aminotransaminase greater than 80 IU/L;
2. kidney dysfunction (serum creatinine greater than 115 mol/L);
3. heart dysfunction;
4. diabetic ketoacidosis or hyperosmolar hyperglycemic non-ketotic coma;
5. psychiatric diseases;
6. chronic disorders requiring medication;
7. current pregnancy; and
8. all patients receiving drugs affecting carbohydrate and lipid metabolism.

Finally, the health status of participants was confirmed by routine biochemical tests.

### 3.4. Treatment

Patients were randomized to receive *Berberis* fruit (1mg, twice daily) or placebo prepared in indistinguishable capsules. The *Berberis* fruit and placebo capsules were provided by Biochemical laboratory.

### 3.5. Clinical and Biochemical Measurements

Patients were visited after an overnight fasting of 10–14 hours. Past medical history of patients was assessed. Patients’ sera were collected at the end of the experimental period to analysis biochemical parameters. Biochemical measurements of serum glucose, lipids, and glycated hemoglobin (HbA1c) were performed. Glucose was measured by glucose oxidase method kit (Pars Azmoon, Tehran, Iran). Serum total cholesterol and triglycerides were measured using standard biochemical kits (Pars Azmoon, Tehran, IR Iran). Blood glycated hemoglobin levels were measured by using Elisa kit (Bioassay technology laboratory, Elisa kit).

### 3.6. Statistical Analysis

Student t-test was used to analysis data before and after the intervention. All results were expressed as mean ± SD. Statistical analysis was performed using SPSS, version 18.0, and P values < 0.05 were considered as statistically significant.

## 4. Results

**Figure 1.**
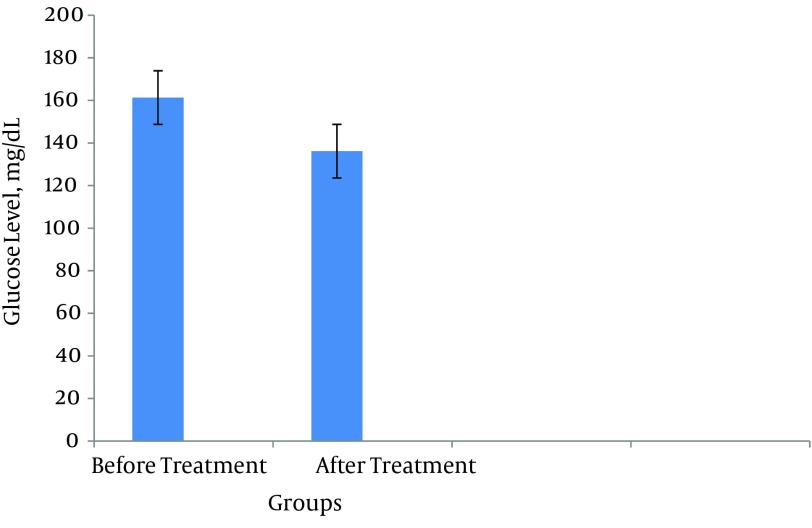
Glucose Level in Patients With Type II Diabetes Data are the mean results of three independent experiments ± SD.

**Figure 2.**
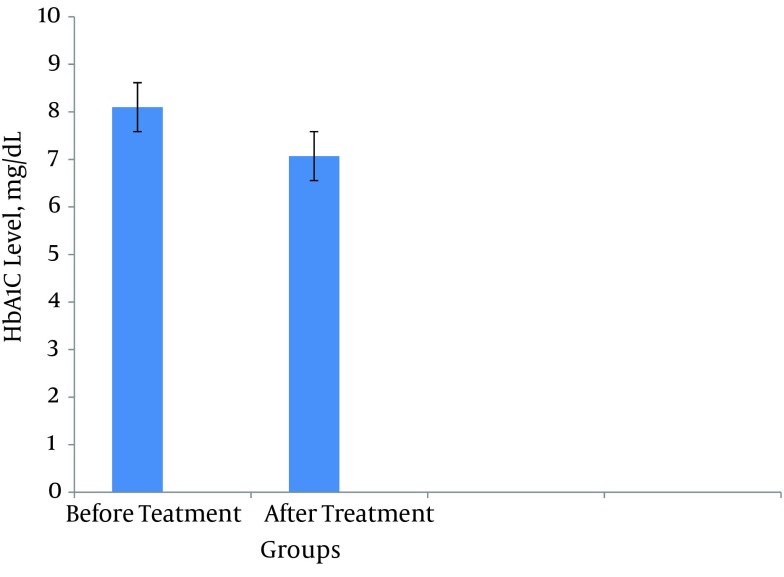
HbA1c Level in Patients With Type II Diabetes Data are the mean results of three independent experiments ± SD, .P < 0.05.

Our results showed significant improvement in glucose level after *Berberis* fruit consumption. This effect of *Berberis* fruit on glucose level may be attributed to achieving a satisfactory regulation of glycemia or changing glucose metabolism. Based on our findings, *Berberis* fruit reduced serum glucose level from 161.31 ± 28.95 mg/dL to 136.15 ± 32.8 mg/dL as shown in Figure 1. In addition, in treatment patients for 8 weeks, Berberine fruit reduced HbA1c from 8.10 ± 1.11 mg/dL to 7.07 ± 1.21 mg/dL in patients with diabetes as shown in Figure 2.

## 5. Discussion

Despite recent advancements in plant medicine, there is still an enormous amount of work needed in this field. In this respect, *Berberis* fruit had a glucose lowering effect at two months in patients with type II diabetes. In addition, *Berberis* fruit decreased HbA1c in patients with diabetes. These results indicated that *Berberis* fruit is a hypoglycemic agent with beneficial effects on glucose metabolism. Given the benefits of Berberine in lowering blood glucose, we speculate that Berberine may be used for patients with type 2 diabetes. Our findings are consistent with those reported elsewhere (3, 4). The precise mechanism of *Berberis* fruit regarding glucose-lowering property has not been fully understood. *Berberis* fruit may facilitate glucose catabolism due to induction of glycolysis pathway in the cell or *Berberis* fruit may inhibit the alpha- glucosidase enzyme (17). We hypothesized that *Berberis* fruit regulates glucose metabolism mechanism by improving glucose tolerance. It may inhibit carbohydrate digestion enzymes activity and decrease carbohydrate digestion process. Therefore, decrease glucose transportation cross the intestinal epithelium and decrease the glucose absorption and finally uptake it. This finding is in agreement with the results obtained by others (4, 5). Further systematic investigations are needed to assess the chemical constituents, pharmacological actions, and toxicity of the *Berberis* fruit to prove its medicinal value. Additional studies are needed to characterize the bioactive compounds responsible for the observed function. *Berberis* fruit value still needs to be determined in human healthcare and detailed information on its usage; duration and dosage must be investigated. Several questions regarding the effect of *Berberis* fruit remain to be answered. In our ongoing research project on medicinal plants, we performed the present study to decrease blood glucose level.

In conclusion, the present study indicated that *Berberis* fruit helps to lower HbA1c, which is critical to control blood glucose level. In addition, *Berberis* fruit has an effect on blood glucose regulation, which might be through activation of carbohydrate metabolism enzymes. Nevertheless, the mechanisms of these encouraging results are not completely clear. To conclude, administration of *Berberis* fruit could attenuate the hyperglycemic state of patients with diabetes. This study had some limitations, first we did not have data on all biochemical factors, and second, our sample was relatively small.
